# Circulating Bilirubin Levels, but Not Their Genetic Determinants, Are Inversely Associated with Steatotic Liver Disease in Adolescents

**DOI:** 10.3390/ijms26072980

**Published:** 2025-03-25

**Authors:** José Patricio Miranda, Juan Cristóbal Gana, Gigliola Alberti, Karen Galindo, Ana Pereira, José Luis Santos

**Affiliations:** 1 Department of Nutrition, Diabetes and Metabolism, School of Medicine, Pontificia Universidad Católica de Chile, Santiago 8331150, Chile; jose.miranda@uc.cl; 2 PhD Program in Epidemiology, Pontificia Universidad Católica de Chile, Santiago 8331150, Chile; 3 Advanced Center for Chronic Diseases (ACCDiS), Pontificia Universidad Católica de Chile & Universidad de Chile, Santiago 8331150, Chile; 4 Department of Pediatric Gastroenterology and Nutrition, Division of Pediatrics, School of Medicine, Pontificia Universidad Católica de Chile, Santiago 8331150, Chile; 5 MSc Program in Nutrition, Pontificia Universidad Católica de Chile, Santiago 8331150, Chile; 6 Instituto de Nutrición y Tecnología de los Alimentos INTA, Universidad de Chile, Macul 7830490, Chile; 7 Department of Health Sciences, Institute for Sustainability and Food Chain Innovation (IS-FOOD), Public University of Navarre, 31006 Pamplona, Spain

**Keywords:** bilirubin, steatotic liver disease, polygenic scores, Native American ancestry, *UGT1A1*

## Abstract

Epidemiologic studies suggest that elevated plasma unconjugated bilirubin confer protection against steatotic liver disease (SLD) in adults. However, evidence supporting this protective role in adolescents remains limited. We aimed to assess the association between serum bilirubin levels and their genetic determinants in protecting against SLD in Chilean adolescents. We conducted a cross-sectional study with 704 adolescents aged 15.4 ± 1 years (52% girls) of the Chilean Growth and Obesity Cohort Study. Ultrasonography echogenicity was used to diagnose SLD. We measured Z-scores of body mass index (z-BMI), total bilirubin (TB), and the genetic determinants of bilirubin (including rs887829 genotypes of *UGT1A1* and bilirubin polygenic scores). Multiple logistic regression models evaluated the associations between standardized TB and its genetic determinants with SLD. We found that 1-SD of standardized plasma TB was significantly associated with a 30% reduction in the likelihood of SLD after adjustment by sex, age, z-BMI, and ethnicity (OR = 0.7; 95% CI = 0.50–0.96; *p* = 0.03). No significant associations were found among the rs887829 genotypes, bilirubin polygenic scores, and SLD in logistic regression models adjusted by covariates. Increased circulating bilirubin levels are unlikely causally associated with protection against SLD, and the cross-sectional association could be due to unmeasured confounding.

## 1. Introduction

For years, bilirubin was considered only the end-product of heme catabolism and a neurotoxic waste compound without any beneficial health effects [1]. The detrimental actions of bilirubin are notoriously represented in kernicterus, which results from extremely high toxic levels of circulating bilirubin in neonates, leading to irreversible brain damage. In contrast, recent evidence suggests that mild constitutive hyperbilirubinemia due to increased unconjugated bilirubin (UCB), as it occurs in Gilbert’s syndrome (GS), may protect against insulin resistance, metabolic syndrome, and liver fat accumulation through multiple mechanisms, including newly discovered endocrine actions attributed to bilirubin [1,2,3].

Bilirubin primarily derives from the degradation of hemoglobin from erythrocytes (80% of total bilirubin production) and other hemoproteins such as myoglobin, cytochromes, and catalases. After roughly 120 days of the erythrocyte functional life, macrophages of the mononuclear phagocyte system in the spleen, bone marrow, and Kupffer cells in the liver engulf senescent erythrocytes and degrade them to release the prosthetic heme group from globin chains [4]. The rate-limiting enzyme of heme catabolism is heme oxygenase (HMOX; two genes: *HMOX1* and *HMOX2*), which opens the porphyrin ring to generate Fe^2+^, CO, and biliverdin, consuming NADPH. Following the HMOX reaction, biliverdin is degraded by biliverdin reductase (*BLVR*; two isoenzymes BLVRA and BLVRB) with the additional consumption of NADPH to produce UCB [4]. UCB is a lipid-soluble compound of yellowish-orange color that requires albumin to travel to the liver via the organic anion transporting polypeptides (OATPs, genes: SLCOs). Once in the hepatocyte, bilirubin is conjugated with glucuronic acid by the UDP-glucuronosyltransferase-A1 (UGT1A1), generating conjugated bilirubin (CB), which is a water-soluble compound that is eliminated in the bile. UGT1A1 is also expressed in enterocytes to produce UCB [5].

The superfamily of UDP-glucuronosyltransferases (UGTs) comprises four families: UGT1, UGT2, UGT3, and UGT8. Among them, the locus of the UGT1A family includes nine functional UGT1A enzymes (UGT1A1, UGT1A3, and UGT1A4-10). Although UGT1A1 metabolizes the conjugation of multiple lipid-soluble toxins, carcinogens, and drugs, this enzyme is the only one among the superfamily of UGTs that conjugates bilirubin in humans [6]. Mutations and gene variants conferring near-total, severe, or partial loss of function in *UGT1A1* lead to increased circulating levels of UCB [7]. The Crigler–Najjar syndrome (CNS) is a rare autosomal disorder caused by near-complete loss-of-function mutations in the *UGT1A1* gene. In contrast, other hypomorphic variants in the *UGT1A1* gene lead to moderate reductions in glucuronidation activity and mild hyperbilirubinemia characteristic of GS. The most frequent gene variant found in GS individuals is a TA insertion in the TATAA box of *UGT1A1* promoter [A(TA)7TAA] (termed UGT1A1*28; instead, the normal TA6), leading to a 50% reduction in gene expression [8]. In Chile and other countries, genome-wide association studies (GWAS) have shown a strong and unique association between *UGT1A1* variants and circulating bilirubin [9,10]. The single nucleotide polymorphisms (SNPs) rs6742078 and rs887829 are the gene variants most significantly associated with hyperbilirubinemia in Chileans, as well as being perfect surrogates of GS mutations [11,12].

Serum bilirubin is commonly measured to differentiate various hepatobiliary conditions/diseases, providing information on the underlying nature of jaundice. The normal reference range of total bilirubin (TB) in serum is 0–1.0 mg/dL, while the range of CB is 0–0.3 mg/dL [13]. Then, TB circulates mostly as UCB bound to albumin and a minor fraction in its conjugated form (CB). It is reported that serum TB levels are reduced in smokers and after eating and increased by prolonged fasting and stress [14]. Compared to men, women of reproductive age show lower levels of circulating TB and UCB because of testosterone down-regulating UGT1A1 [15,16]. In Chile, TB > 1.4 mg/dL was nine times more common in male than in female adults, underscoring the influence of gender in determining circulating bilirubin [12]. Additionally, serum TB levels decline with age during young adulthood and middle age [2,17] but increase in older people [18,19,20]. Individuals with gene variants causing GS show serum TB of 1.0–5 mg/dL, slightly above normal TB values. There is no universal definition of GS, despite that all of them are necessarily based on *UGT1A1* genotypes or/and serum TB levels of >1 mg/dL, with normal values of liver enzymes ALT/AST. Applying different criteria to Chile, the point estimates of the prevalence of GS in Chile are 11% (considering only *UGT1A1* homozygous genotypes), 8.3% (TB > 1.0 mg/dL), or 2.6% (TB > 1.4 mg/dL) [11,12].

Several studies have proposed a potential protective effect of mild hyperbilirubinemia on cardiovascular risk and related comorbidities [1,2,3], partially attributed to the recognized potent antioxidant properties of bilirubin [21,22] and the resulting inhibition of reactive oxygen species generation [23]. It is also reported that circulating bilirubin shows important anti-inflammatory capacity, with serum TB levels inversely associated with total white cell count (a simple marker of chronic inflammation) and the inhibition of pro-inflammatory cytokines [24,25,26,27]. Moreover, it was proposed that bilirubin may act as a hormone stimulating the nuclear receptor PPARα to enhance insulin sensitivity, liver ketogenesis, fatty acid oxidation, and fibroblast growth factor-21 (FGF21) expression [28,29,30,31]. Interestingly, the humanized mouse that contains the genetic variant HuUGT*28, which is analogous to the UGT1A1*28 mutation causing GS in humans [32], represents a genuine animal model for GS, given that it displays similar levels of mild constitutive UCB hyperbilirubinemia compared to GS subjects. The HuUGT*28 mice showed sharp protective actions against hyperglycemia and insulin resistance in response to high-fat diets, as evaluated in glycemic responses to an intraperitoneal glucose tolerance test and through glycemic measurements across the mice’s adult life. Moreover, the HuUGT*28 mouse is also protected against high-fat-diet-induced steatosis, as evidenced by the reduced fat content of liver sections compared to control mice. Regarding observational studies in humans, multiple epidemiologic cohorts reported an inverse association between serum TB levels and insulin resistance surrogates, lower prevalence of metabolic syndrome (MetS), type 2 diabetes (T2D), and steatotic liver disease (SLD) [33,34,35,36,37,38,39,40,41]. The study of the protective actions of bilirubin is important since it may provide new therapy approaches given that the exogenous administration of bilirubin nanoparticles has been proposed to prevent MetS and SLD in mice [31,42,43].

SLD is defined by the accumulation of fat exceeding 5% of the total hepatocytes content. We have reported that the prevalence of SLD has increased significantly and is closely associated with the increasing prevalence of obesity in Chilean adolescents from the Growth and Obesity Cohort Study (GOCS) [44,45,46]. Evidence from epidemiologic studies suggests that plasma bilirubin may protect against adult SLD. However, the proposed protective role of bilirubin against SLD in adolescents is limited. Herein, we cross-sectionally evaluated the possible protective role of bilirubin in developing SLD in adolescents from the GOCS cohort, including biochemical measurements of circulating TB and its genetic determinants related to *UGT1A1* gene variants or polygenic scores.

## 2. Results

### 2.1. Description of the GOCS Participants

Table 1 shows the general characteristics of GOCS participants according to sex and SLD status. The mean age of the 704 participants was 15.4 ± 1 year, being 51.7% girls. The overall prevalence of SLD in the GOCS cohort determined by ultrasonography was 9.4%, without differences by sex (9.1% in boys and 9.6% in girls). In general, median serum bilirubin levels were 45% higher in boys than in girls (*p* = 1.65 × 10^−16^) and significantly lower among SLD cases versus controls, both in boys and in girls (*p* < 0.02). Regarding anthropometry, participants with SLD showed a higher z-BMI and greater waist circumference than controls in both boys and girls (*p* < 0.001 for each). When comparing the biochemical measurements, we found that the SLD participants also had significantly higher serum levels of the liver enzymes ALT, AST, and GGT in both boys and girls (*p* < 0.032 for each). There were no significant differences in the composition of global ancestry NAT, EUR, and AFR when comparing SLD cases with controls and by sex (*p* > 0.1 for each).

### 2.2. Observational Associations Between Serum Total Bilirubin and SLD

In the whole cohort, an increase of 1-SD in the IRNT-TB was associated with a 30% reduction in the odds ratio for SLD (OR = 0.7; *p* = 0.03; 95% CI = 0.50–0.96) (Table 2). When we evaluated IRNT-TB by quintiles, we found a dose-response effect with an increase in protection as the quintile increases; however, this association was near significant in Q4 and Q5 only (OR = 0.40 and OR = 0.37, *p* = 0.06, respectively). After stratification by sex, we found a significant association in girls for Q4 (OR = 0.17, *p* = 0.02) and a near-significant association for boys in Q5 (OR = 0.26, *p* = 0.09).

### 2.3. Association Between Genetic Determinants of Bilirubin and SLD

The gene variant rs887829 (346 bp upstream *UGT1A1*) is in Hardy–Weinberg equilibrium (*p* = 0.8) and its frequency in GOCS participants was 32.5% for the minor allele T, which is in line with what was described for the admixed Latino population in the 1000 Genomes project (37.9%), and gnomAD (31.8%) (https://www.ncbi.nlm.nih.gov/snp/, accessed on 3 January 2025). We did not find a significant association between genotypes of the rs887829 variant and SLD in crude models or adjusting by age, sex, z-BMI, and 5 PCs (Table 3) (*p* > 0.15 for each).

The polygenic scores PGS000697, PGS001942, and PGS002160 were estimated with more than 255,000 participants from the UK Biobank, for predominantly European, African, and Asian populations. Between 1159 and 120,068 variants were used for their construction, which were covered over 99% in GOCS participants (Supplementary Table S1). The correlation between TB and PGSs varied between 0.22 and 0.61 in the population where they were described, being stronger among individuals from the European population.

When evaluating the three PGSs in GOCS, we found a’s moderately significant correlation with serum TB levels, for the cohort, boys, and girls (Spearman ρ = 0.50–0.52, ρ = 0.59–0.60, and ρ = 0.45–0.47, respectively) (all *p* < 0.001) (Supplementary Figure S1). However, we did not find significant associations between the PGSs and SLD in crude models or after the adjustment of covariates (Table 4).

## 3. Discussion

It has been proposed that bilirubin may have a protective role in cardiometabolic diseases given its antioxidant/anti-inflammatory properties, but that it also acts as an endocrine factor enhancing insulin sensitivity, as well as promoting liver fatty acid oxidation, ketone body production, and FGF21 expression, leading to a reduction in liver fat accumulation [1,2,27,30,47]. The proposed endocrine effects of bilirubin are possibly mediated by the stimulation of the nuclear receptor PPARα in the liver, adipose tissue, and cardiac muscle [2,28,29,30,31,32]. In addition to PPARα stimulation, additional metabolic effects of bilirubin may derive from its binding to other receptors such as the Mas-related G-protein coupled receptor member X4 (MRGPRX4) and Aryl hydrocarbon receptor (AHR) [31,48]. Animal studies and epidemiological observations support a role of bilirubin as a protective factor in SLD [33,34,35,36,37,38,39,40,41,42,49,50,51,52,53]. Interestingly, it was reported that high plasma bilirubin is a distinctive feature of the metabolically healthy obesity phenotype, considering obesity as the most important risk factor of SLD [54]. Another piece of evidence supporting the protective role of bilirubin in cardiometabolic diseases derives from drugs affecting UGT1A1 activity. It is reported that atazanavir (HIV-1 protease inhibitor used in the treatment of HIV infection) inhibits UGT1A1 activity, while phenobarbital (a barbiturate antiepileptic agent) induces it [55]. A double-blind placebo-controlled cross-over trial using a 3-day atazanavir treatment in patients with type 2 diabetes induced mild hyperbilirubinemia similar to GS individuals achieving TB levels from 0.4 mg/dL (basal) to 3.8 mg/dL at the end of the intervention. In this trial, atazanavir intervention was strongly associated with an enhanced plasma antioxidant capacity and improvements in endothelial function [56]. It has also been reported that individuals living with HIV infection under treatment with atazanavir and hyperbilirubinemia show significant reductions in type 2 myocardial infarction (a common infarction type in HIV patients derived from an oxygen demand-supply mismatch) [57].

Our observational study found a significant inverse association between serum TB levels and SLD in the GOCS cohort of Chilean adolescents, suggesting a potential protective role of bilirubin in preventing SLD. Similar results were described in a cohort of Danish children and adolescents [58]. These results also align with observations from adult cohorts of Asian or North American origin and meta-analysis [42,49,50,51,52,53,59]. However, evidence from observational studies for the association between bilirubin and SLD is also conflicting since it was found that participants with moderate and severe steatosis had significantly higher levels of TB compared with controls in adults from the Chinese Han population [53]. One possibility is that such direct association (instead of inverse) is generated by a different mechanism, such as cholestatic liver disease increasing TB (especially direct bilirubin) or by an alteration of the thyroid hormone function affecting bilirubin metabolism. In this sense, triiodothyronine (T3) plays a crucial role in liver metabolism, influencing both the synthesis and degradation of fatty acids and cholesterol. Studies have shown that patients with SLD have higher circulating levels of thyroid-stimulating hormone (TSH) and both free and total T3, compared to those without SLD. This alteration in T3 levels may also be related to changes in the conversion of thyroxine (T4) to T3 in the liver, which may be compromised in the presence of SLD [60,61]. Furthermore, prospective studies suggest that normal-to-high T3 levels may predict an increased risk of incidence SLD in adults [60,62]. Interestingly, T3 specifically decreases *UGT1A1* expression in a dose-dependent manner in rat hepatocyte cultures [63,64], possibly leading to increased circulating unconjugated bilirubin levels and mild hyperbilirubinemia. Although partially mimicking what occurs in GS, simultaneous increased T3 and bilirubin may not be protective against SLD in this scenario, opening new therapeutic approaches to prevent SLD through exogenous tissue-specific modulation of thyroid hormones targeting the hepatocyte. Recent studies show that the administration of low doses of levothyroxine (LT4) could be effective in reducing the prevalence of SLD, both in patients with subclinical hypothyroidism and in euthyroid T2D patients [65,66]. Also promising are the results with resmetirom (MGL-3196), a selective thyroid hormone β-receptor agonist that became the first approved drug to manage SLD [67,68].

Due to the possibility that the protective observational associations described for bilirubin are derived from unmeasured confounding, we included a genetic instrumental variable to reduce this possible bias in the results. In previous studies, we reported that the variant rs887829, close to *UGT1A1*, was the most strongly associated with serum bilirubin levels in Chileans and in participants with a Native American ancestry component of the Chilean population [11], explaining 37.6% of the TB variance. Although we do not have information on adults of Native American origin, other studies have reported that the variance explained by variants near *UGT1A1* is close to 20% in the US population, and decreases to close to 10% in the Asian population [49,50]. The GOCS cohort has an admixed Latino ancestry, with 45.6% being of Native American (predominantly Mapuche) origin, 52.5% being European, and a low proportion being of African ancestry, averaging 1.9%. Therefore, analyzing SLD or other variables only allows us to know the effect of combined ancestry.

In contrast to the potential protective associations found for TB in reducing liver steatosis, we also found that the primary genetic determinant of serum TB (rs887829 of *UGT1A1* gene) was not significantly associated with SLD in Chileans. Again, similar results were reported from children and adolescents from Denmark, with no significant associations between *UGT1A1* genotypes and SLD [58]. The PREVEND study also found a significant association between the rs6742078 variant (in high linkage disequilibrium with the rs887829 variant) and serum TB, without finding significant associations between *UGT1A1* genotypes and SLD [50]. In addition to *UGT1A1* genotypes, we also used, in our study, polygenic scores (PS) for serum bilirubin termed PGS000697, PGS001942, and PGS002160 (https://www.pgscatalog.org/, accessed on 3 January 2025) to capture a greater proportion of the circulating TB variance [69,70]. Although all three PGSs were reported in the multi-ethnic UK Biobank, we validated them in our cohort of admixed Latino ancestry, finding a significant moderate correlation between serum TB and such genetic scores. Similar to using *UGT1A1* genotypes alone, we did not find significant associations between GOCS-estimated bilirubin PGSs and SLD risk. Despite the lack of significant association among the variant near *UGT1A1*, the PGSs, and SLD risk, we cannot rule out that these results are due to reduced statistical power in our study.

In observational epidemiology, it is assumed that causal inference regarding associations between metabolic/environmental variables and the risk for a disease is negatively affected by unmeasured or residual confounding as well as reverse causation effects. A commonly used strategy for disentangling causal from non-causal effects in the relation between a risk/protective factor (in this case, circulating bilirubin) and a disease (in this case, SLD) is the use of Mendelian randomization (MR) studies [71,72]. In this study design, genetic variants are used as instrumental proxies for exposures of interest, with the final purpose of establishing the causal relation between a given exposure and a health-related outcome. We believe that it would be interesting to perform a formal MR study in the GOCS cohort; however, we estimate that the statistical power is only 0.28 when considering a sample size of 704, alpha 0.05, proportion of cases 0.09, OR per standard deviation of the exposure 0.71, and proportion of variance explained for the association between the rs887829 variant and bilirubin 0.37 (https://shiny.cnsgenomics.com/mRnd/, accessed on 3 January 2025). Based on this information, to achieve a statistical power of 0.8, a sample size of at least 2900 participants would be necessary to perform an MR study. Such studies have been conducted in adults from the Han Chinese population and in adults from the European population in the PREVEND study [49,50]. Although both studies concluded a lack of evidence supporting a causal association between higher bilirubin levels and protection against SLD, both lack sufficient statistical power to rule out bilirubin as a key molecule in the pathophysiology of SLD.

## 4. Materials and Methods

### 4.1. Study Design

Cross-sectional analysis was performed within the “Growth and Obesity Chilean Cohort Study” (GOCS). This cohort was initiated in 2006 and comprised 1196 children aged 2.6–4.0 years who attended public nursery schools in the Metropolitan Region of Santiago [73]. During adolescence, GOCS participants were invited to a follow-up to determine the presence of steatotic liver disease (SLD) by ultrasonography, to which 784 responded. Concurrently, we also obtained genome-wide genotypes in 950 participants from DNA isolated from leukocytes (see below). The present study included 704 participants with both SLD determinations and genomic analyses (age 15.4 ± 1 years; 340 males, 364 females; see detailed description below). Exclusion criteria included participants with previous liver damage and alcohol abuse. This study was approved by the Ethics Review Board of the School of Medicine, Pontificia Universidad Católica de Chile (Santiago, Chile). Written informed consent from parents or guardians and children’s assent were obtained for GOCS participants.

### 4.2. Evaluation of Steatotic Liver Disease (SLD)

The diagnosis of SLD was determined by an increase in the echogenicity of the liver compared to the renal cortex. Two experienced pediatric radiologists evaluated SLD using ultrasonography (Acuson S2000, 6-2 MHz convex and 9-4 MHz linear transducers; Munich, Germany) [46].

### 4.3. Anthropometry

The weight and height of GOCS participants were measured with a digital scale Tanita BC-418 (Tokyo, Japan) and a portable stadiometer (SECA 222; Hamburg, Germany), respectively. We calculate the body mass index (BMI) as the ratio between weight (in kg) and height squared (in mt^2^). Then, we calculated z-scores of BMI for age and sex (z-BMI) following the standards recommended by the WHO [74]. Using a diameter tape (Lufkin W606PM, Cleveland, OH, USA), we measured the waist circumference under the iliac crest after expiration.

### 4.4. Serum Biochemical Determinations

Total serum bilirubin levels (TB) were determined by the classical Diazo method and used as a surrogate for unconjugated bilirubin levels (UCB). Alkaline phosphatase (ALP), alanine aminotransferase (ALT), aspartate aminotransferase (AST), and gamma-glutamyltransferase (GGT) activity were measured in the central laboratory UC-Christus. All biochemical measurements were performed on a Cobas C System (Roche, Basel, Switzerland).

### 4.5. Genotyping of GOCS Participants

Genotypes of the rs887829 variant, a surrogate marker of the gene variant causal of Gilbert’s syndrome in the *UGT1A1* gene, as well as genome-wide SNPs, were obtained from the Infinium^®^ Multi-Ethnic Global BeadChip (Illumina, Inc.; San Diego, CA, USA; >1.7 million common and rare variants across the genome). Details of the genotyping quality control and SNPs determination in GOCS participants were described elsewhere [11,75,76,77].

### 4.6. Estimation of Bilirubin Polygenic Scores in GOCS

Polygenic scores (PGSs) for TB termed PGS000697 [69], PGS001942 [70], and PGS002160 [70], reported in adults of the UK Biobank, were calculated in GOCS participants from the Michigan Imputation Server using the above-mentioned genotyping data [78].

### 4.7. Estimation of Ancestry and Genetic Principal Components in GOCS

The estimate of the proportion of global Native American (NAT), European (EUR), or African (AFR) ancestry (ethnicity) was obtained from the weighted sum of local ancestry for each chromosome (1 to 22), obtained with RfMix v2 software for each participant as previously described [11,79]. Population global ancestry was inferred using Plink v1.9, after removing genetic regions in high linkage disequilibrium, sex chromosome variants, and pruned genotypes using an independent pairwise approach with a window size of 50 kb, a step size of 5 SNPs, and r^2^ cutoff threshold of 0.2. We estimated 20 genetic principal components of ethnicity (PCs) to account for population stratification [11,76,80]. Because the admixed Chilean population has a genetic heritage primarily derived from Europeans, Native Americans, and, to a lesser extent, Africans, we have estimated that 5 PCs are sufficient to account for this genetic variability [11,76].

### 4.8. Statistical Analysis

We performed inverse rank-based normal transformation (IRNT) of TB to normalize data (mean = 0; standard deviation = 1) and used multiple logistic regression models to assess the association between this transformed variable and SLD status in the GOCS cohort. Models were adjusted for age, sex, z-BMI, and 5 Principal Components (PCs). In the whole cohort and after sex stratification, we also used multiple logistic regression to assess associations between quintiles (Q1–Q5) of IRNT-TB, *UGT1A1* rs887829 genotypes, or bilirubin PGSs with SLD. We again adjusted these models for age, z-BMI, and 5 PCs. We used Spearman’s correlation to assess the association between circulating TB in GOCS participants and the PGSs. Statistical analyses were carried out using RStudio v2023.06.1, and Stata BE v17.0.

## 5. Conclusions

Our observation study found a significant inverse association between serum bilirubin and SLD in Chilean adolescents. However, no association was found between established genetic determinants of circulating bilirubin (rs887829 variant of *UGT1A1* and PGSs) and SLD. Then, increased circulating bilirubin levels are unlikely to be causally associated with protection against SLD, given the lack of association between instrumental genetic variables of serum bilirubin and the disease. The recapitulation of our results and other published studies suggests that the observational association between TB and SLD might still derive from unmeasured confounding.

## Figures and Tables

**Table 1:** Characteristics of the steatotic liver disease cases and controls of the GOCS cohort.

	Boys (N = 340)			Girls (N = 364)		
	Controls (N = 309) Mean (SD) or Median (IQR)	SLD Cases (N = 31) Mean (SD) or Median (IQR)	*p* Value	Controls (N = 329) Mean (SD) or Median (IQR)	SLD Cases (N = 35) Mean (SD) or Median (IQR)	*p* Value
*Age and anthropometry*						
Age (years)	14.94 (0.92)	14.83 (0.82)	0.476	15.78 (0.86)	15.70 (0.90)	0.61
z-score of BMI	0.47 (1.13)	2.15 (0.86)	**<0.001**	0.77 (0.99)	2.14 (1.19)	**<0.001**
Waist circumference (cm)	74.04 (9.16)	92.06 (11.94)	**<0.001**	73.60 (8.74)	88.19 (16.34)	**<0.001**
*Circulating metabolite and enzyme measurements*						
TB (mg/dL)	0.47 (0.35–0.7)	0.45 (0.31–0.56)	**0.019**	0.33 (0.24–0.47)	0.26 (0.19–0.35)	**0.009**
ALP (U/L)	195 (145–250)	191 (142.5–233)	0.532	89 (77–101)	95 (84.5–112.5)	0.096
ALT (U/L)	16 (12–20)	23 (17–29.5)	**<0.001**	17 (14–19)	19 (16–20.5)	**0.032**
AST (U/L)	18 (14–20)	21 (17–34)	**0.003**	13 (10–17)	16 (13–24.5)	**0.015**
GGT (U/L)	12 (10–14)	16 (13–20.5)	**0.001**	10 (8–13)	14 (10.5–18)	**0.008**
*Global ancestry proportions*						
NAT	0.45 (0.10)	0.49 (0.11)	0.098	0.45 (0.09)	0.46 (0.07)	0.505
EUR	0.53 (0.10)	0.49 (0.11)	0.110	0.53 (0.09)	0.52 (0.07)	0.464
AFR	0.02 (0.01)	0.02 (0.01)	0.178	0.02 (0.01)	0.02 (0.01)	0.546

IQR: Interquartile range; TB: total bilirubin; ALP: alkaline phosphatase; ALT: alanine aminotransferase; AST: aspartate aminotransferase; GGT: gamma glutamyltransferase; NAT: Native American ancestry; EUR: European ancestry; AFR: African ancestry. In bold, significant associations.

**Table 2:** Odds ratios for steatotic liver disease occurrence by standardized total serum bilirubin in the GOCS cohort.

	Cohort (N = 704)		Boys (N = 340)		Girls (N = 364)	
Continuous IRNT-TB or by Quintile *	OR_adj_ (95% CI)	*p* Value	OR_adj_ (95% CI)	*p* Value	OR_adj_ (95% CI)	*p* Value
Continuous	**0.70 (0.50–0.96)**	**0.03**	0.68 (0.40–1.12)	0.14	0.70 (0.45–1.07)	0.10
Q1	*ref*	*-*	*ref*	*-*	*ref*	*-*
Q2	0.73 (0.31–1.69)	0.47	0.51 (0.16–1.98)	0.34	0.53 (0.15–1.72)	0.30
Q3	0.48 (0.18–1.21)	0.13	0.86 (0.24–2.94)	0.81	1.09 (0.35–3.40)	0.88
Q4	0.40 (0.15–1.01)	0.06	0.32 (0.07–1.21)	0.10	**0.17 (0.03–0.70)**	**0.02**
Q5	0.37 (0.13–1.00)	0.06	0.26 (0.05–1.12)	0.09	0.41 (0.10–1.49)	0.19

Cohort OR_adj_: adjusted by sex, age, z-BMI, and 5 PCs. Boys/Girls OR_adj_: adjusted by age, z-BMI, and 5 PCs. IRNT-TB: inverse-ranked normal transformed total bilirubin. * Quintiles were estimated for each group independently. In bold, significant associations. ORs are expressed by 1-SD of IRNT-TB.

**Table 3:** Odds ratios for steatotic liver disease occurrence by genotypes of the variant rs887829 near *UGT1A1* in the GOCS cohort.

	Genotypes	OR_adj_ (95% CI)	*p* Value
Cohort	CC (N = 319)	*ref*	*-*
	CT (N = 312)	1.40 (0.76–2.62)	0.28
	TT (N = 73)	0.75 (0.25–2.01)	0.59
Boys	CC (N = 143)	*ref*	*-*
	CT (N = 152)	2.05 (0.79–5.72)	0.15
	TT (N = 45)	0.52 (0.09–2.35)	0.42
Girls	CC (N = 176)	*ref*	*-*
	CT (N = 160)	1.00 (0.43–2.31)	0.99
	TT (N = 28)	0.98 (0.21–3.56)	0.98

Cohort OR_adj_: adjusted by sex, age, z-BMI, and 5 PCs. Boys/Girls OR_adj_: adjusted by age, z-BMI, and 5 PCs.

**Table 4:** Risk of steatotic liver disease occurrence by three different polygenic scores of total bilirubin in the GOCS cohort.

	PGS000697		PGS001942		PGS002160	
Continuous PGSs or by Quintiles *	OR_adj_ (95% CI)	*p* Value	OR_adj_ (95% CI)	*p* Value	OR_adj_ (95% CI)	*p* Value
**Cohort (N = 704)**						
Continuous	1.23 (0.29–5.09)	0.77	1.00 (0.24–4.01)	1.00	1.28 (0.32–5.11)	0.72
Q1	*ref*	*-*	*ref*	*-*	*ref*	*-*
Q2	0.70 (0.26–1.87)	0.47	0.94 (0.36–2.48)	0.90	0.80 (0.30–2.13)	0.66
Q3	1.55 (0.60–4.08)	0.36	1.55 (0.63–3.91)	0.34	1.05 (0.39–2.80)	0.93
Q4	0.99 (0.39–2.55)	0.99	0.66 (0.23–1.83)	0.43	0.94 (0.36–2.48)	0.90
Q5	1.03 (0.41–2.61)	0.96	1.18 (0.48–2.94)	0.72	1.01 (0.41–2.59)	0.98
**Boys (N = 340)**						
Continuous	1.05 (0.12–8.95)	0.96	0.94 (0.11–7.65)	0.95	1.55 (0.18–13.3)	0.69
Q1	*ref*	*-*	*ref*	*-*	*ref*	*-*
Q2	0.70 (0.11–3.91)	0.69	0.37 (0.06–2.01)	0.27	0.68 (0.13–3.51)	0.64
Q3	1.70 (0.40–8.02)	0.48	1.62 (0.42–6.78)	0.49	1.32 (0.30–6.30)	0.71
Q4	2.06 (0.54–9.04)	0.30	1.45 (0.38–5.91)	0.58	1.86 (0.47–8.37)	0.38
Q5	0.88 (0.19–1.58)	0.86	0.63 (0.15–2.73)	0.53	0.75 (0.16–3.66)	0.72
**Girls (N = 364)**						
Continuous	1.09 (0.14–7.73)	0.93	0.86 (0.12–5.73)	0.87	0.93 (0.13–5.98)	0.94
Q1	*ref*	*-*	*ref*	*-*	*ref*	*-*
Q2	1.09 (0.39–3.11)	0.87	0.69 (0.23–1.99)	0.50	1.64 (0.57–5.12)	0.37
Q3	0.60 (0.17–1.90)	0.39	0.74 (0.25–2.13)	0.58	0.84 (0.23–2.93)	0.78
Q4	0.48 (0.12–1.62)	0.25	0.19 (0.03–0.79)	0.04	0.61 (0.15–2.26)	0.47
Q5	1.16 (0.41–3.28)	0.78	1.19 (0.44–3.22)	0.73	1.89 (0.65–5.90)	0.25

Cohort OR_adj_: adjusted by sex, age, z-BMI, and 5 PCs. Boys/Girls OR_adj_: adjusted by age, z-BMI, and 5 PCs. * Quintiles were estimated for cohort, boys, and girls independently.

## Data Availability

All the information associated with this work is found throughout the text and in the Supplemental Materials.

## Supplementary Materials

The following supporting information can be downloaded at: https://www.mdpi.com/article/10.3390/ijms26072980/s1.
