# Supplementary material for: Circulating Bilirubin Levels, but Not Their Genetic Determinants, Are Inversely Associated with Steatotic Liver Disease in Adolescents
Source: Int J Mol Sci. 2025 Mar 25;26(7):2980. doi: 10.3390/ijms26072980 (PMC11988633; doi:10.3390/ijms26072980)

SUPPLEMENTARY MATERIAL

Circulating Bilirubin Levels, but Not Their Genetic Determi-nants, Are Inversely Associated with Steatotic Liver Disease in Adolescents

José Patricio Miranda 1,2,3, Juan Cristóbal Gana 4, Gigliola Alberti 4, Karen Galindo 5, Ana Pereira 6 and José Luis San-tos 1,2,7,*

1 Department of Nutrition, Diabetes and Metabolism, School of Medicine, Pontificia Universidad Católica de Chile, Santiago 8331150, Chile

2 PhD Program in Epidemiology, Pontificia Universidad Católica de Chile, Santiago 8331150, Chile

3 Advanced Center for Chronic Diseases (ACCDiS), Pontificia Universidad Católica de Chile & Univer-sidad de Chile, Santiago 8331150, Chile

4 Department of Pediatric Gastroenterology and Nutrition, Division of Pediatrics, School of Medicine, Pontificia Universidad Católica de Chile, Santiago 8331150, Chile

5 MSc Program in Nutrition, Pontificia Universidad Católica de Chile, Santiago 8331150, Chile

6 Instituto de Nutrición y Tecnología de los Alimentos INTA, Universidad de Chile, Macul 7830490, Chile

7 Department of Health Sciences, Institute for Sustainability and Food Chain Innovation (IS-FOOD), Public University of Navarre, 31006 Pamplona, Spain

* Correspondence: jsantosm@uc.cl

Supplementary Table S1. Characteristics of the total bilirubin PGSs described in the UK Biobank and the coverage obtained in the GOCS cohort.

	**PGS000697**	**PGS001942**	**PGS002160**
Individuals for score development/training	255,256	391,124	391,124
Variants in the PGS	1,159	33,190	120,068
Variants covered in GOCS	1,148 (99.05%)	33,190 (100%)	120,068 (100%)
Spearman's correlation to bilirubin by ancestry in the described population			
European	0.44 (N=63,531)	0.61 (N=18,919)	0.60 (N=18,919)
African	0.39 (N=5,978)	0.55 (N=3,640)	0.47 (N=3,640)
South Asian	0.44 (N=7,313)	0.58 (N=5,983)	0.57 (N=5,983)
East Asian	0.22 (N=1,069)	0.41 (N=1,702)	0.40 (N=1,702)

Supplementary Figure S1. Correlation of total serum bilirubin levels and the calculated polygenic risk scores of total bilirubin in the GOCS Cohort. Spearman's correlation (ρ) was estimated according to sex ($p<0.001$ for each).

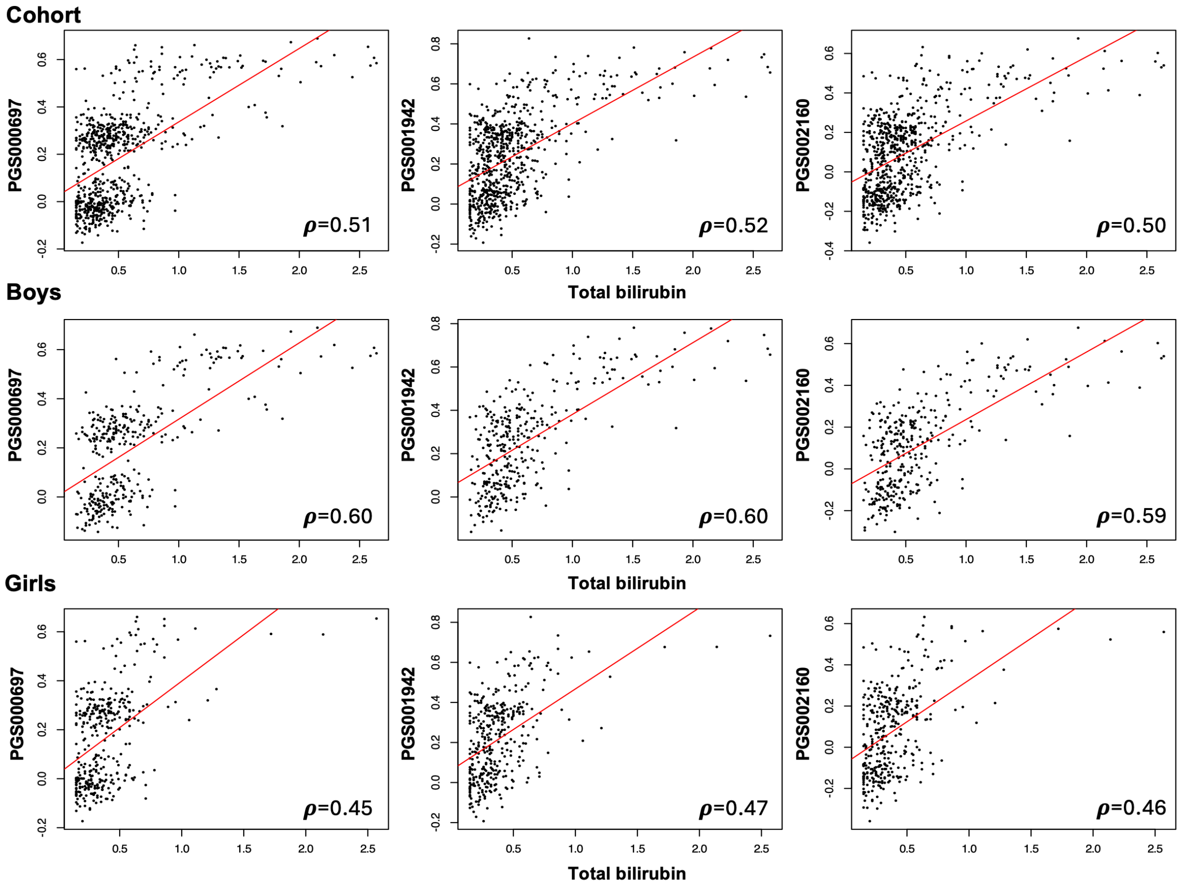

Supplement: Supplementary file 1 [file ijms-26-02980-s001.zip › ijms-3482892-supplementary.pdf]
